# Potentially Important Aroma Compounds in Dark Tea and Volatile Compound Changes During Primary Pile Fermentation: A Systematic Review and Meta‐Analysis

**DOI:** 10.1002/fsn3.72499

**Published:** 2026-10-28

**Authors:** Shu Gong, Jinwei Xu, Guizhen Tang, Binyu Mao, Qinmei Zhang, Haimin Chen

**Affiliations:** ^1^ Zhejiang Institute of Economics and Trade Hangzhou China; ^2^ Tea Research Institute, the Chinese Academy of Agricultural Sciences Hangzhou China; ^3^ College of Life Sciences and Medicine Zhejiang Sci‐Tech University Hangzhou China

**Keywords:** dark tea, meta‐analysis, odor activity value, pile fermentation, systematic review, volatile compounds

## Abstract

Pile fermentation is essential to the characteristic aroma of dark tea, yet studies have reported inconsistent changes in individual volatile compounds, leaving unclear which compounds change consistently and retain potential odor relevance after fermentation. We systematically searched six English‐ and Chinese‐language databases and included 11 studies of primary solid‐state pile fermentation of dark tea. Within‐study relative concentration percentiles and odor activity values (OAVs) were used to characterize post‐fermentation relative abundance and potential odor contribution. Random‐effects meta‐analysis was applied to comparisons quantifiable both before and after fermentation, whereas detection‐state transitions were summarized separately for zero and non‐detect data. Eighteen compounds met the prespecified criterion for potentially important aroma compounds. After Benjamini–Hochberg false‐discovery‐rate correction, 10 compounds increased significantly and one decreased significantly. Detection‐state evidence further showed that methoxy‐substituted aromatic compounds predominantly changed from non‐detectable or non‐quantifiable before fermentation to quantifiable after fermentation. Integrating the three evidence streams identified alpha‐ionone, terpinen‐4‐ol, (E)‐2‐octenal, 1,2‐dimethoxybenzene, 1,2,3‐trimethoxybenzene, and 1,2,4‐trimethoxybenzene as fermentation‐enriched potentially important aroma compounds, whereas linalool was fermentation‐depleted but retained potential odor relevance after fermentation. This cross‐study synthesis identifies compound‐level patterns shared across dark‐tea pile‐fermentation systems and provides prioritized candidates for targeted quantification and sensory validation.

## Graphical abstract

Pile fermentation drives the transformation of volatile profiles in dark tea. Systematic review and OAV consistency analysis reveal key aroma‐active compounds and their contributions to characteristic sensory attributes, including woody, aged, herbal, fatty, and floral/sweet notes.

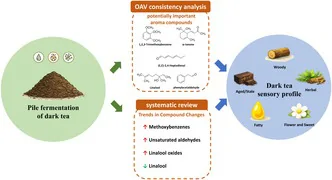

## 1 Introduction

Dark tea is a distinctive category of microbially fermented tea produced in China and includes ripened Pu‐erh tea, Liupao tea, Fu brick tea, Qingzhuan tea, and Tianjian tea. Pile fermentation is central to the development of dark‐tea quality. Under warm, humid, and aerated conditions, endogenous reactions and microbial metabolism jointly alter the color, aroma, and taste of the raw material, progressively generating complex aged, woody, fungal, and sweet notes (Lin et al. 2021; L. Wang et al. 2025). Dark‐tea volatiles encompass alcohols, aldehydes, ketones, esters, hydrocarbons, oxygen‐containing heterocycles, and methoxy‐substituted aromatic compounds (L. Wang et al. 2025; Z. Yang et al. 2013). During pile fermentation, hydrolysis of glycosidically bound precursors, lipid oxidation, carotenoid cleavage, and transformations of amino acids and carbohydrates occur concurrently, increasing some constituents while depleting others and thereby restructuring the volatile profile (Guo et al. 2025; Hu et al. 2021; Z. Yang et al. 2013).

Gas chromatography–mass spectrometry (GC–MS), gas chromatography‐olfactometry (GC‐O), electronic‐nose analysis, and multi‐omics approaches have demonstrated substantial changes in the diversity and composition of aroma compounds during dark‐tea processing. However, variation in tea type and raw material, pile‐fermentation conditions and endpoints, microbial communities, extraction procedures, and analytical platforms often yields discordant or even opposing trajectories for the same compound (Hu et al. 2021; Q. Li et al. 2020; P. Liu et al. 2022; Ma et al. 2021; W. Zhang, Cao, et al. 2021). Existing reviews have largely summarized volatile composition, sensory characteristics, and putative formation mechanisms (Lin et al. 2021; L. Wang et al. 2025), but cross‐study evidence remains insufficient to resolve two questions: which aroma compounds show directionally consistent changes across tea materials and fermentation conditions, and which post‐fermentation compounds consistently reach odor activity values (OAV) ≥ 1 across studies and may therefore contribute materially to dark‐tea aroma?

To address these questions, we conducted a systematic review and meta‐analysis of volatile aroma changes during primary solid‐state pile fermentation of dark tea. To our knowledge, this is the first cross‐study synthesis to integrate three complementary evidence streams: concentration changes for compounds quantifiable before and after fermentation, summarized as response ratios (RRs); detection‐state transitions for comparisons containing zeros or non‐detects (ND), summarized without arbitrary positive‐value imputation; and post‐fermentation odor relevance, evaluated from within‐study relative concentration rankings and the cross‐study consistency of OAV ≥ 1 (T. Li, Wei, et al. 2024; Y. Li, Wang, et al. 2024; Pang et al. 2019). This framework was designed to identify fermentation‐enriched and fermentation‐depleted compounds with consistent trajectories, and to distinguish these changes from compounds that remain potentially odor‐relevant after fermentation, without pooling heterogeneous absolute concentrations or OAV magnitudes.

## 2 Methods

### 2.1 Study Design and Literature Search Strategy

This systematic review and meta‐analysis evaluated changes in volatile compounds from the beginning to the completion of primary pile fermentation of Chinese dark tea. The review was conducted and reported in accordance with the Preferred Reporting Items for Systematic Reviews and Meta‐Analyses (PRISMA) 2020 statement (Page et al. 2021). Because the review concerns a food‐processing operation rather than a clinical or health intervention, it was not eligible for prospective registration in the principal clinical‐review registries. Retrospective registration was not undertaken after screening and extraction had commenced because it would not constitute prospective protocol registration. To preserve transparency, the complete eligibility criteria, database‐specific search strategies, data‐processing rules, and PRISMA checklist are provided in the Data S1.

Web of Science, PubMed, Embase, Scopus, China National Knowledge Infrastructure (CNKI), and Wanfang Data were searched from database inception to 18 August 2026, without language restrictions. Search strategies combined three concept groups: dark tea, fermentation, and volatile compounds or aroma. The complete database‐specific queries, search fields, platforms, limits, and search logs are provided in the Data S1. Reference lists of eligible articles and relevant reviews were also examined, and studies recommended during peer review were checked through supplementary manual searches.

All records were merged and deduplicated first by digital object identifier (DOI), and then, when a DOI was unavailable, by normalized title, author, and publication year. Two reviewers (Shu Gong and Jinwei Xu) independently screened titles and abstracts and assessed the full texts of potentially eligible reports. Records whose titles or abstracts clearly described inoculated fermentation or another ineligible process were excluded at title‐and‐abstract screening; records with insufficient information proceeded to full‐text assessment. Disagreements were resolved by discussion and, when necessary, consultation with a third reviewer (Qinmei Zhang).

### 2.2 Eligibility Criteria

#### 2.2.1 Inclusion Criteria

Studies were eligible when all of the following criteria were met: (A) an original, peer‐reviewed experimental study with an accessible full text and reliably extractable numerical data; (B) investigation of primary solid‐state pile fermentation of Chinese dark tea under natural, traditional, or industrial conditions, or an uninoculated laboratory fermentation comparable to primary pile fermentation in substrate, processing purpose, and endpoint; (C) use of green tea or another tea material that had not previously undergone the target pile‐fermentation step as the starting substrate; (D) clear identification of the sample collected before pile fermentation and the endpoint sample defined by the original authors as completion of the first pile‐fermentation process; (E) compound‐level quantitative concentrations obtained using an internal standard, external standard, calibration curve, or another explicitly calibrated method; and (F) availability for both pre‐ and post‐fermentation groups of means, standard deviations or reconstructable replicate measurements, and sample sizes.

#### 2.2.2 Exclusion Criteria

Studies were excluded if any of the following applied: (A) the product or process could not be classified as primary pile fermentation of dark tea, or the starting material was already a post‐fermented dark‐tea product; (B) fermentation was conducted in tea infusion, extract, or another liquid culture rather than with solid tea leaves as the substrate; (C) the study investigated artificial inoculation with a single strain or defined consortium, exogenous‐assisted fermentation, storage, aging, secondary fermentation, or re‐fermentation after primary pile fermentation; (D) a comparison between the pre‐fermentation (or day‐0) sample and the completed pile‐fermentation endpoint could not be extracted, or only one processing stage was reported; (E) volatile compounds were reported only qualitatively or as raw peak areas, relative peak areas, peak‐area percentages, or normalized signals rather than extractable quantitative concentrations; (F) means, dispersion measures, sample sizes, or other numerical information required for quantitative synthesis could not be reconstructed from the article or Data S1; or (G) the report was a review, conference abstract, patent, book chapter, editorial, non‐peer‐reviewed document, or a duplicate report of the same experiment for which a more complete report was available.

### 2.3 Data Extraction

Two reviewers independently extracted data using a standardized form. Recorded study characteristics included first author, publication year, dark‐tea type, fermentation mode, substrate, definitions of the starting and endpoint samples, fermentation duration, sample matrix, extraction and instrumental methods, quantitative calibration procedure, concentration unit, pre‐ and post‐fermentation means, standard deviations, sample sizes, replicate type, compound name, Chemical Abstracts Service (CAS) number, and detection state. Data in the main text and Data S1 were cross‐checked. Discrepancies were resolved by returning to the original report and reaching consensus.

For studies reporting multiple processing stages, the principal comparison was defined as that between the pre‐fermentation or Day 0 sample and the endpoint designated by the authors as completion of primary pile fermentation; intermediate stages were not treated as independent principal effects. If a study contained both an eligible natural‐fermentation treatment and ineligible inoculated or exogenous‐addition treatments, only the eligible arm was extracted.

### 2.4 Data Cleaning and Classification of Detection States

Before synthesis, compound names and CAS numbers were checked manually against the original reports. Synonyms referring to the same chemical entity were consolidated under one canonical name and CAS number. Pre‐ and post‐fermentation data within a study had to share the same, or a reliably convertible, unit, matrix, and quantitative basis. Values reported as 0, 0+/−0, not detected (n.d.), or ND were classified as NDs.

### 2.5 Post‐Fermentation Concentration and Odor‐Activity Analyses

Absolute post‐fermentation concentrations were not pooled because the included studies differed in sample matrix, extraction system, concentration unit, and quantification method. Units were harmonized only within the same measurement dimension: mg/L was converted to micrograms/L and micrograms/g to micrograms/kg, whereas mass and volume concentrations were not interconverted. To describe the relative position of each compound within the volatile profile reported by an individual study arm, post‐fermentation concentrations were converted to within‐study relative concentration percentiles. This approach follows the use of within‐sample percentile ranks in cross‐platform data integration to reduce differences in scale and measurement platform (Cui et al. 2017):

$$P_{\mathrm{ij}}=\frac{{\bar{r}}_{\mathrm{ij}}-1}{m_{i}-1}\times 100$$

where mean rank *r̄*_*ij*_ is the mean rank assigned to compound *j* in study arm *i*, and *m*_*i*_ is the total number of post‐fermentation concentration records in that study arm. Tied values were assigned their mean rank; zero and non‐detect values were assigned to the lowest concentration group. For compounds reported by at least five independent study arms, the median percentile, interquartile range, and minimum‐to‐maximum range were summarized. These percentiles were used solely for descriptive ranking and were not interpreted as pooled concentrations or effect sizes. The complete within‐study concentration percentile summaries are provided in Table S7.

Odor Activity Was Evaluated Only for Post‐Fermentation Samples. For Compound j in Study i, the Study‐Level Odor Activity Value Was Calculated as

$${\mathrm{OAV}}_{\mathrm{ij}}=\frac{C_{\text{after},\mathrm{ij}}}{{\mathrm{OT}}_{j}}$$

where *C*_after,*ij*_ is the post‐fermentation concentration and OT_*j*_ is the selected odor threshold in water. Thresholds explicitly defined by chemical entity, matrix, and unit in the original studies were prioritized; the compilations of van Gemert and the Leibniz‐LSB@TUM Odorant Database were used as supplementary sources (Kreissl et al. 2022; van Gemert 2011). Candidate thresholds were individually checked for chemical identity, CAS number, isomer, matrix, unit, and source traceability. Absolute OAVs from different studies or matrices were not pooled. Instead, the number and proportion of studies in which post‐fermentation OAV was ≥ 1 were summarized for each compound, the underlying concentration, detection‐state, and OAV records are provided in Table S8. A compound was defined as a potentially important aroma compound when it was evaluated in at least five studies and had OAV ≥ 1 in at least 80% of them. The cross‐study OAV consistency results are summarized in Table S9.

### 2.6 Meta‐Analysis and Integration of Evidence

Comparisons were classified as quantifiable‐to‐quantifiable (Q‐to‐Q), non‐detect‐to‐quantifiable (ND‐to‐Q), quantifiable‐to‐non‐detect (Q‐to‐ND), or non‐detect‐to‐non‐detect (ND‐to‐ND). Data were then separated according to whether the conditions for calculating the natural logarithm of the response ratio (lnRR) were met. Q‐to‐Q comparisons with positive means before and after fermentation were used for effect‐size estimation and meta‐analysis. Comparisons involving a zero or non‐detect were not numerically imputed and were excluded from lnRR calculation; they were summarized only in the descriptive detection‐state analysis.

#### 2.6.1 Meta‐Analysis

For Q‐to‐Q comparisons with positive means before and after fermentation, concentration change was expressed as the natural logarithm of the response ratio (Hedges et al. 1999):

$${\text{lnRR}}_{i}=\ln\left(\frac{{\bar{X}}_{\text{after},i}}{{\bar{X}}_{\text{before},i}}\right)$$

The sampling variance was approximated from the group means, standard deviations, and sample sizes as:

$$v_{i}=\frac{{{\mathrm{SD}}_{\text{after},i}}^{2}}{n_{\text{after},i}{{\bar{X}}_{\text{after},i}}^{2}}+\frac{{{\mathrm{SD}}_{\text{before},i}}^{2}}{n_{\text{before},i}{{\bar{X}}_{\text{before},i}}^{2}}$$

Only comparisons with a positive sampling variance entered inverse‐variance analysis. For each Q‐to‐Q comparison i, ${\bar{X}}_{\text{after},i}$ and ${\bar{X}}_{\text{before},i}$ denote the post‐ and pre‐fermentation group means, SD_after,*i*_ and SD_before,*i*_ the corresponding standard deviations, and *n*_after,*i*_ and *n*_before,*i*_ the group sample sizes. For compounds with at least three independent Q‐to‐Q comparisons, lnRRs were pooled using a DerSimonian‐Laird random‐effects model (DerSimonian and Laird 1986) and back‐transformed to RRs with 95% confidence intervals (CIs) and two‐sided *p* values. RR > 1 indicated a higher post‐fermentation concentration and RR < 1 a lower concentration. Heterogeneity was assessed using Cochran's Q test and quantified by *I* ^2^ and *τ* ^2^.

The two‐sided *p* values from all compound‐level meta‐analyses constituted one multiple‐testing family and were adjusted using the Benjamini–Hochberg procedure (Benjamini and Hochberg 1995). Statistical significance was defined as *q* < 0.05. Compounds with significant adjusted results and RR > 1 were classified as fermentation‐enriched compounds supported by quantitative evidence; those with RR < 1 were classified as fermentation‐depleted compounds. Compounds with a directional effect that did not survive false‐discovery‐rate correction were reported only as possible trends. Analyses were conducted in Stata 14.0 (StataCorp, College Station, TX, USA).

#### 2.6.2 Detection‐State Analysis

Comparisons in which at least one group was zero or non‐detectable were not assigned a positive replacement value and were not used to calculate lnRR. They were classified as ND‐to‐Q, Q‐to‐ND, or ND‐to‐ND; Q‐to‐Q counts were retained to show the distribution of all comparison states. For each compound, the number and proportion of independent studies or study arms in each state were tabulated. ND‐to‐Q denoted a transition from non‐detectable before fermentation to quantifiable after fermentation, Q‐to‐ND the reverse transition, and ND‐to‐ND non‐detection at both time points.

ND‐to‐Q and Q‐to‐ND were considered directional detection‐state transitions. A compound was regarded as showing a consistent fermentation‐related state trend when directional transitions occurred in at least three independent studies or study arms and at least 80% were in the same direction. Compounds dominated by ND‐to‐Q transitions were classified as fermentation‐enriched compounds supported by detection‐state evidence, whereas those dominated by Q‐to‐ND transitions were classified as fermentation‐depleted compounds. Compounds with fewer than three directional transitions or less than 80% directional agreement were described without assigning a consistent trend.

Detection‐state analysis was descriptive and did not yield a pooled effect size, *p* value, or false‐discovery‐rate‐adjusted value. When meta‐analysis and detection‐state analysis both met their respective criteria and pointed in the same direction, the compound was considered to have both quantitative and detection‐state support. When only one evidence stream met its criterion, the source of evidence was specified. Discordant evidence was reported separately rather than forced into a single category.

### 2.7 Methodological Quality Assessment

Two reviewers independently assessed methodological quality using a review‐specific framework developed for before‐and‐after food‐processing studies. Domains comprised the appropriateness of study design and tea‐sample selection, clarity of the pre‐fermentation and endpoint definitions, independence and reporting of replicates, use of calibrated quantification, availability of uncertainty data, uniqueness of compound identity, and traceability of extracted data. Each domain was rated as low risk or some concerns, followed by an overall study‐level judgment. Overall study quality and eligibility of individual records for meta‐analysis were evaluated separately. Disagreements were resolved by consensus or consultation with a third reviewer. No automated tools were used for the final judgments.

## 3 Results

### 3.1 Study Selection and Characteristics

The six database searches identified 1416 records: 284 from Web of Science, 137 from PubMed, 120 from Embase, 306 from Scopus, 197 from CNKI, 372 from Wanfang Data, and 12 additional records were examined through manual searching; all were duplicates of database records or were excluded before entry into the unique‐record screening flow. After 572 duplicate database records were removed, 856 records underwent title‐and‐abstract screening; 806 were excluded, leaving 50 reports for full‐text eligibility assessment (Figure 1).

**FIGURE 1:**
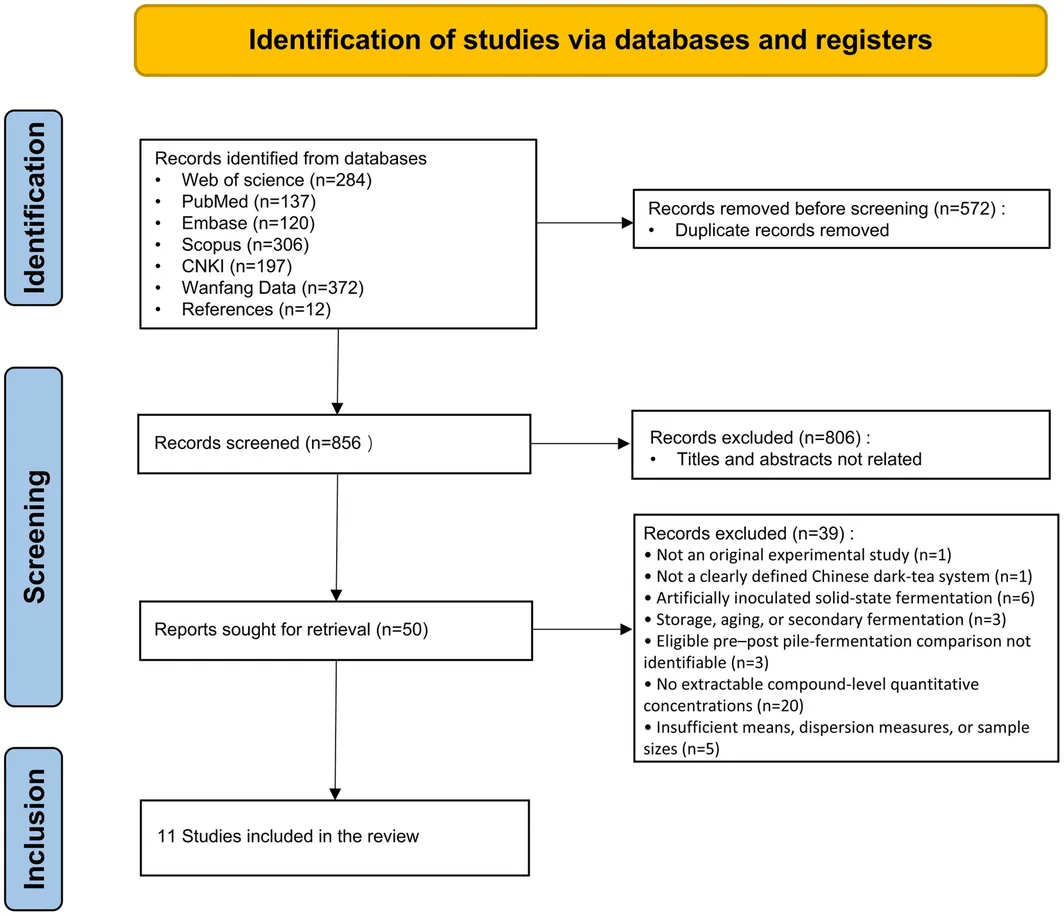
PRISMA flow diagram of study identification, screening, full‐text assessment, and inclusion.

Thirty‐nine reports were excluded at full‐text assessment: one was not an original experimental study, one did not investigate a clearly defined Chinese dark‐tea system, six primarily examined artificially inoculated solid‐state fermentation, three concerned storage, aging, or secondary fermentation after primary pile fermentation, three did not permit identification of the comparison between the beginning and completion of primary pile fermentation, 20 did not provide extractable compound‐level quantitative concentrations, and five lacked the means, reconstructable dispersion measures, or sample sizes required for quantitative comparison. Eleven studies were included in the quantitative synthesis (Table 1).

**TABLE 1:** Characteristics of the studies included in the quantitative synthesis.

Study	Tea type	Matrix	Eligible comparison	Duration/endpoint	Analytical method	Quantification	Replicates (*n*)	Data source
Huo et al. (2024)	Liupao tea	Dry tea	Natural pile: 0–30 h	30 h	HS‐SPME‐GC–MS	Internal‐standard semi‐quantification	3, independent biological replicates	Main‐text Table 4
Y. Li et al. (2022)	Dark raw tea	Dry tea	Natural pile: Day 0–Day 30	30 days	HS‐SPME‐GC–MS	Internal‐standard semi‐quantification	3, independent biological replicates	Article and numerical tables
Hu et al. (2021)	Dark raw tea	Dry tea	Natural pile: S0–S6	37 days	HS‐SPME‐GC–MS	Internal‐standard semi‐quantification	3, independent biological replicates	Article and Data S1
Li, Wei, et al. (2024)	Ripened Pu‐erh tea	Dry tea	Spontaneous pile arm: SDGT‐B‐PF to SPPF‐5	38 days	GC–MS/GC‐O	Internal−/external‐standard quantification	3, independent biological replicates	Main‐text Table 2
Wen et al. (2023)	Dark tea	Tea infusion/extract	Natural pile: pre‐ to post‐fermentation	Not reported/post‐pile‐fermentation sample	GCxGC‐QTOFMS	Internal‐standard semi‐quantification	3, independent biological replicates	Article and Data S1
Fang et al. (2024)	Qingzhuan tea	Dry tea	Primary pile segment: SLS to PLS	33 days	HS‐SPME‐GC–MS	Internal‐standard semi‐quantification	3, independent biological replicates	Article and Data S1
Weng et al. (2026)	Ripened Pu‐erh tea	Dry tea	Natural pile: RM to RP	45 days	HS‐SPME‐GC–MS	Internal−/external‐standard quantification	3, replicate‐level data	Table S1
Wu et al. (2026)	Primary dark raw tea	Tea infusion/extract	Natural primary pile: 0 h–48 h	48 h	GC–MS	External‐standard quantification	4, independent biological replicates	Table S3
Q. Li et al. (2020)	Fu brick tea	Dry tea	Natural pile segment: S1–S2	12 h	HS‐SPME‐GC–MS	Calibration‐curve quantification	3, independent batches	Main‐text table 1
W. Zhang, Cao, et al. (2021)	Yinghong No. 9 dark tea	Dry tea	Natural pile: Day 0–Day 60	60 days	HS‐SPME‐GC–MS	Internal‐standard semi‐quantification	6, independent biological replicates	Table S4
P. Liu et al. (2022)	Qingzhuan tea	Tea infusion/extract	Primary pile segment: pre‐ to post‐pile	30 days	GC–MS/GC‐O	Internal‐standard semi‐quantification	3, independent biological replicates	Article and Data S1

The included studies covered Liupao tea, ripened Pu‐erh tea, Qingzhuan tea, Fu brick tea, and other dark teas produced by natural or uninoculated primary pile fermentation. All studies reported compound‐level quantitative data derived from calibrated or internal‐standard‐based methods but differed in matrix, extraction system, concentration unit, fermentation duration, and analytical platform. In total, 595 post‐fermentation compound records were extracted from the 11 studies.

Three studies were judged to be at low methodological risk and eight to raise some concerns; none was judged to be at high risk. No study was excluded solely on the basis of the quality assessment.

### 3.2 Post‐Fermentation Concentration Distribution and Odor Activity

Because matrices, extraction systems, concentration units, and quantification procedures varied across studies, absolute post‐fermentation concentrations were not pooled. Concentrations were instead converted within each study arm to relative concentration percentiles to describe the position of each compound in the reported volatile profile. The 11 studies contributed 595 post‐fermentation concentration records; 37 compounds were reported by at least five independent study arms and entered the primary concentration‐distribution summary (Figure 2a; Table S7).

**FIGURE 2:**
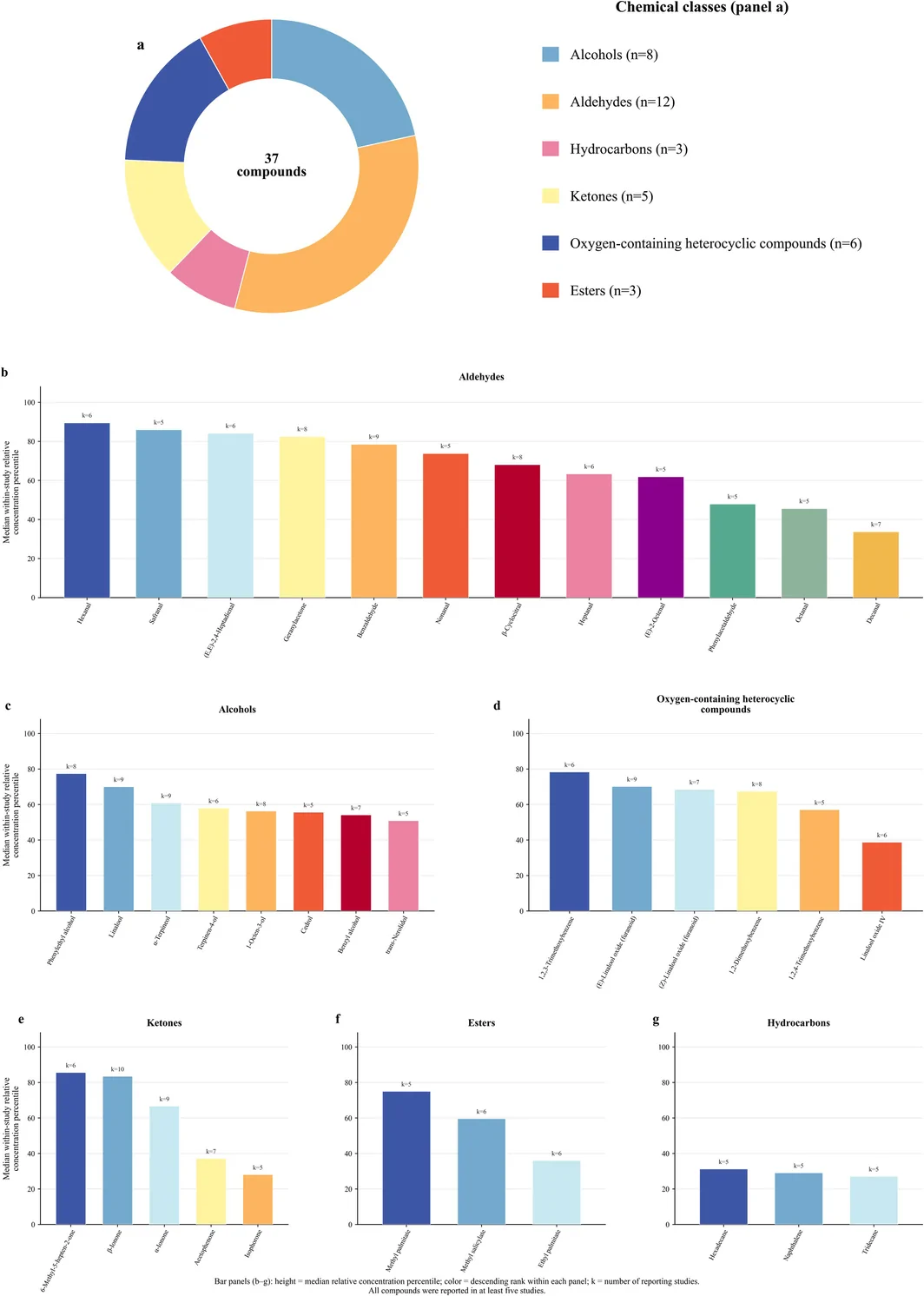
Post‐fermentation within‐study relative concentration percentiles of 37 volatile compounds reported by at least five independent study arms. (a) Distribution by chemical class; (b–g) median percentiles for each compound class. Higher percentiles indicate higher relative concentrations within individual study arms. *k* denotes the number of reporting study arms.

The 10 compounds with the highest median within‐study relative concentration percentiles were hexanal (89.6), safranal (86.0), 6‐methyl‐5‐hepten‐2‐one (85.6), (E,E)‐2,4‐heptadienal (84.1), beta‐ionone (83.7), geranylacetone (83.3), benzaldehyde (78.9), 1,2,3‐trimethoxybenzene (78.4), phenethyl alcohol (77.5), and methyl palmitate (75.0) (Figure 2b–g). The minimum percentiles of (E,E)‐2,4‐heptadienal, beta‐ionone, and 1,2,3‐trimethoxybenzene remained 46.4, 51.1, and 53.5, respectively, indicating relatively stable positions across studies. By contrast, the ranges for hexanal, safranal, 6‐methyl‐5‐hepten‐2‐one, and phenethyl alcohol were wider. These percentiles describe relative rank within individual volatile profiles; they are not pooled concentrations or effect sizes.

Of the 595 post‐fermentation records, 294 (49.4%) could be matched to a verified odor threshold and represented 59 compounds; 191 of these records (65.0%) had OAV ≥ 1 The underlying concentration, detection‐state, and OAV records are provided in Table S8. Eighteen compounds met the prespecified consistency criterion of evaluation in at least five studies and OAV ≥ 1 in at least 80% of them, representing 30.5% of the threshold‐evaluable compounds (Figure 3; Table S9). Beta‐ionone, benzaldehyde, alpha‐ionone, 1,2‐dimethoxybenzene, (E,E)‐2,4‐heptadienal, 1,2,3‐trimethoxybenzene, heptanal, terpinen‐4‐ol, (E)‐2‐octenal, 1,2,4‐trimethoxybenzene, cedrol, naphthalene, nonanal, and octanal had OAV ≥ 1 in every evaluable study. The corresponding proportions were 88.9% for linalool, 87.5% for 1‐octen‐3‐ol, 83.3% for hexanal, and 80.0% for phenylacetaldehyde.

**FIGURE 3:**
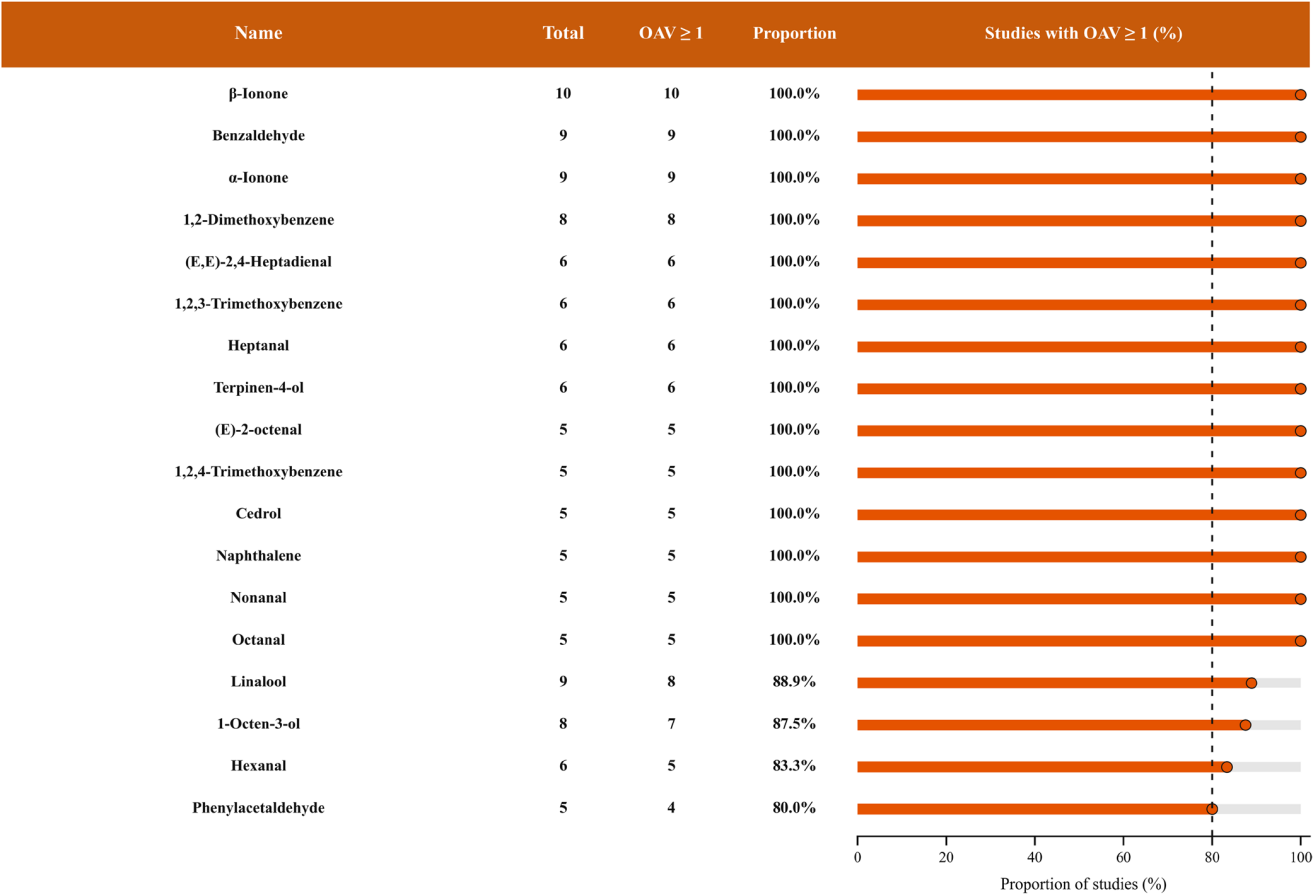
Cross‐study consistency of post‐fermentation OAV ≥ 1. The figure includes compounds evaluated in at least five studies with OAV ≥ 1 in at least 80% of studies. The dashed line indicates the prespecified 80% threshold. OAV, odor activity value.

Aldehydes constituted the largest candidate class (eight compounds) (Figure 2b). Hexanal had the highest median relative concentration percentile (89.6) and OAV ≥ 1 in five of six studies. Benzaldehyde reached OAV ≥ 1 in all nine studies in which it was evaluable; (E,E)‐2,4‐heptadienal and heptanal did so in all six, and (E)‐2‐octenal, nonanal, and octanal in all five. Phenylacetaldehyde met the threshold in four of five studies. The reported odor descriptors were almond‐like and fruity for benzaldehyde, fatty for (E,E)‐2,4‐heptadienal, fresh for heptanal and (E)‐2‐octenal, fatty and citrus‐like for nonanal, waxy and citrus‐like for octanal, honey‐like and floral for phenylacetaldehyde, and green or grassy for hexanal. Thus, aldehydes provided potential green, fresh, fatty, waxy, citrus‐like, fruity, and honey‐like/floral dimensions to the post‐fermentation aroma profile.

Alcohol candidates comprised terpinen‐4‐ol, cedrol, linalool, and 1‐octen‐3‐ol (Figure 2c). Linalool had a median percentile of 70.4 and OAV ≥ 1 in eight of nine studies. Terpinen‐4‐ol and cedrol met the threshold in all six and five evaluable studies, respectively, and 1‐octen‐3‐ol in seven of eight. Terpinen‐4‐ol and cedrol were associated mainly with woody notes, linalool with floral and fruity notes, and 1‐octen‐3‐ol with a characteristic mushroom‐like note. Phenethyl alcohol, despite a high median percentile of 77.5, had OAV ≥ 1 in only three of eight studies and did not meet the consistency criterion, demonstrating that relative concentration rank and odor activity provide distinct evidence.

The three methoxy‐substituted aromatic compounds also showed strong cross‐study OAV consistency (Figure 2d). Median percentiles were 67.7 for 1,2‐dimethoxybenzene, 78.4 for 1,2,3‐trimethoxybenzene, and 57.1 for 1,2,4‐trimethoxybenzene; all evaluable studies (eight, six, and five, respectively) had OAV ≥ 1. All three compounds have been associated with the stale dimension characteristic of post‐fermented dark tea.

Both beta‐ionone and alpha‐ionone, which are characterized by violet‐like and floral notes, had OAV ≥ 1 in all evaluable studies, with median percentiles of 83.7 and 66.7 (Figure 2e). In comparison, 6‐methyl‐5‐hepten‐2‐one had a high median percentile (85.6) but reached OAV ≥ 1 in only three of six studies and was not classified as a potentially important aroma compound. Naphthalene, described as pungent, was the only hydrocarbon to meet the candidate criterion: its median percentile was only 29.1, but OAV was ≥ 1 in all five evaluable studies (Figure 2g). Conversely, safranal, geranylacetone, and methyl palmitate ranked highly by relative concentration but had OAV ≥ 1 in only 60.0%, 62.5%, and 0% of evaluable studies, respectively. Relative abundance and odor activity therefore captured complementary, non‐interchangeable features.

Taken together, the 18 compounds represented complementary odor dimensions rather than a single dominant note. The three methoxy‐substituted aromatic compounds supported a stale background; terpinen‐4‐ol and cedrol contributed woody notes; the ionones, linalool, benzaldehyde, and phenylacetaldehyde supplied violet‐like, floral, fruity, almond‐like, and honey‐like attributes; the aliphatic aldehydes added green, fresh, fatty, waxy, and citrus‐like nuances; and 1‐octen‐3‐ol contributed a mushroom‐like note. Naphthalene added a pungent dimension in the threshold‐based screening, although its origin and sensory relevance require verification. The characteristic post‐fermentation aroma of dark tea may therefore arise from an aged/stale and woody foundation overlaid by floral/violet, fruity/honey‐like, green, fatty/waxy, citrus‐like, and mushroom‐like nuances. This interpretation describes the odor dimensions represented by the OAV‐screened candidates and does not substitute for sensory reconstruction or omission testing.

### 3.3 Changes in Volatile Compounds During Pile Fermentation

#### 3.3.1 Meta‐Analysis of Q‐To‐Q Comparisons

After data cleaning, 49 compounds had at least three independent Q‐to‐Q comparisons and were analyzed using separate DerSimonian‐Laird random‐effects models. Thirteen compounds had an unadjusted *p* < 0.05; 11 remained significant after Benjamini‐Hochberg correction, including 10 that increased and linalool, which decreased after pile fermentation (Figure 4). Under the prespecified definitions, these were classified as quantitatively supported fermentation‐enriched or fermentation‐depleted compounds.

**FIGURE 4:**
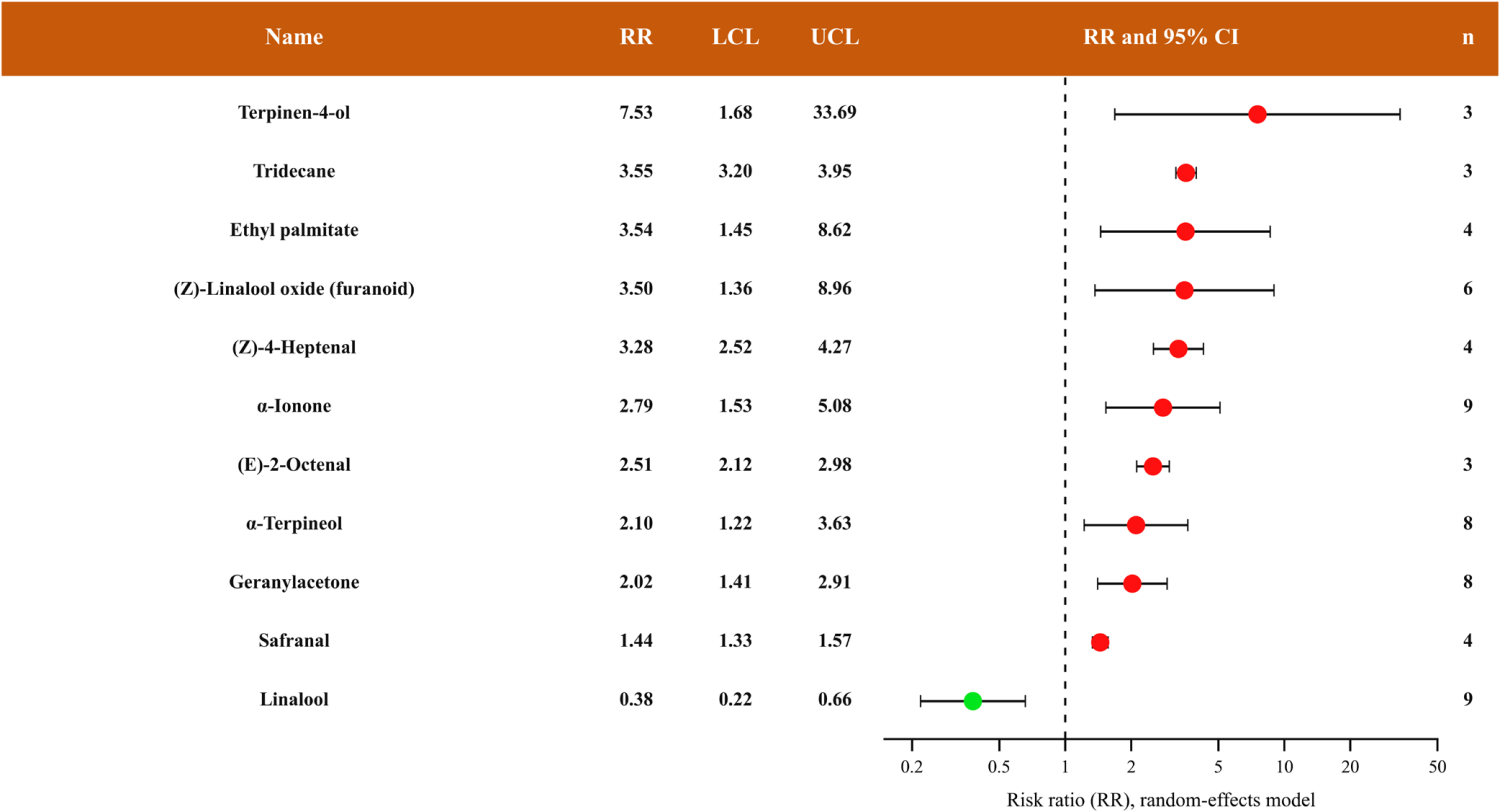
DerSimonian–Laird random‐effects meta‐analysis of 11 volatile compounds significant after Benjamini–Hochberg false‐discovery‐rate correction. Points and horizontal lines represent pooled response ratios (RRs) and 95% confidence intervals, respectively. The dashed line indicates RR = 1; *n* denotes the number of Q → Q comparisons.

Changes were particularly marked among terpene alcohols and their oxidation products. Terpinen‐4‐ol showed the largest pooled increase (RR = 7.53, 95% CI 1.68–33.69), and all three Q‐to‐Q comparisons increased. Alpha‐terpineol also increased (RR = 2.10, 95% CI 1.22–3.63), with seven of eight comparisons in the same direction, as did (Z)‐linalool oxide (furanoid) (RR = 3.50, 95% CI 1.36–8.96). All three were classified as fermentation‐enriched.

Linalool was the only compound that decreased significantly after false‐discovery‐rate correction. Its post‐fermentation concentration was approximately 38% of the pre‐fermentation concentration (RR = 0.38, 95% CI 0.22–0.66), and eight of nine comparisons decreased; it was therefore classified as fermentation‐depleted. The decrease in linalool alongside increases in selected oxygenated derivatives indicates that related terpene alcohols do not change synchronously during pile fermentation.

Among aldehydes, all four Q‐to‐Q comparisons for (Z)‐4‐heptenal increased (RR = 3.28, 95% CI 2.52–4.27), as did all three comparisons for (E)‐2‐octenal (RR = 2.51, 95% CI 2.12–2.98). Safranal increased more modestly but consistently across four comparisons (RR = 1.44, 95% CI 1.33–1.57). These three aldehydes met the criterion for fermentation enrichment.

Other significantly increased compounds were tridecane, ethyl palmitate, alpha‐ionone, and geranylacetone. The pooled RRs were 3.55 (95% CI 3.20–3.95) for tridecane and 3.54 (95% CI 1.45–8.62) for ethyl palmitate, with every Q‐to‐Q comparison increasing. Alpha‐ionone and geranylacetone increased to 2.79‐fold (95% CI 1.53–5.08) and 2.02‐fold (95% CI 1.41–2.91) of their pre‐fermentation concentrations; seven of nine and seven of eight comparisons, respectively, increased.

Acetophenone and beta‐ionone also had unadjusted *p* < 0.05, with pooled RRs of 3.22 (95% CI 1.10–9.39) and 1.62 (95% CI 1.07–2.44), respectively. Their adjusted *q* values were 0.122 and 0.087; however, neither met the prespecified significance criterion; they were therefore reported only as possible upward trends.

Between‐study heterogeneity was generally high: 45 of the 49 meta‐analyses had *I* ^2^ ≥ 75%. Among the 11 significant compounds, heterogeneity was low for tridecane and safranal (*I* ^2^ = 0 and 4.8%, respectively), moderate for (E)‐2‐octenal (*I* ^2^ = 53.6%), and high for the remaining eight (*I* ^2^ = 87.9%–99.5%). The fermentation‐enriched and fermentation‐depleted classifications therefore describe the cross‐study direction and statistical evidence, whereas pooled RRs should not be interpreted as fixed fold changes applicable to every dark‐tea type or production condition.

#### 3.3.2 Detection‐State Transitions Before and After Fermentation

To capture changes that lnRR cannot represent, all 595 comparisons were also classified by detection state: 441 (74.1%) were Q‐to‐Q, 105 (17.6%) ND‐to‐Q, 37 (6.2%) Q‐to‐ND, and 12 (2.0%) ND‐to‐ND (Figure 5). ND‐to‐Q transitions were 2.8 times as frequent as Q‐to‐ND transitions, indicating that records newly detected or quantifiable after fermentation outnumbered those becoming non‐detectable.

**FIGURE 5:**
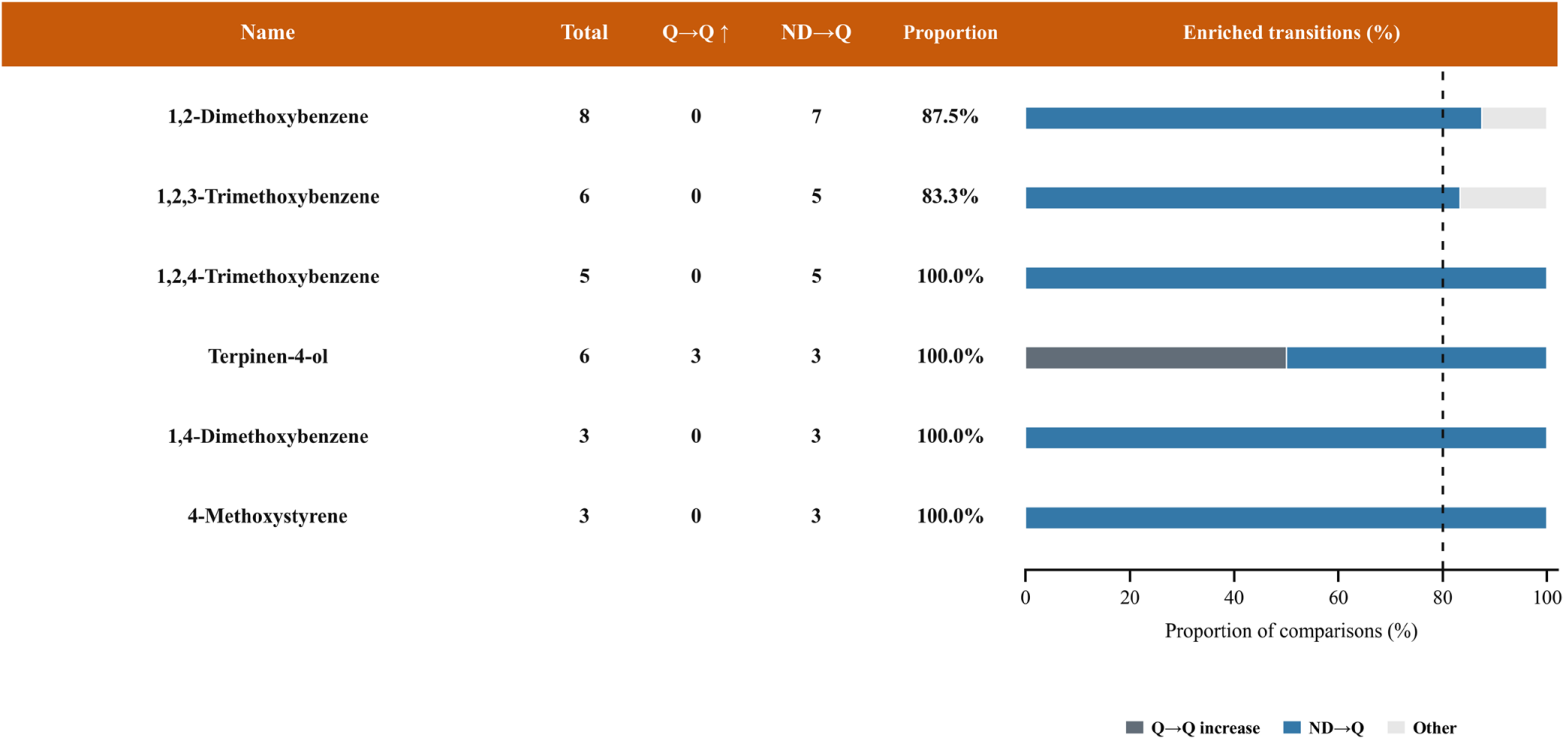
Quantitative and detection‐state evidence supporting fermentation enrichment. Dark gray indicates increasing Q → Q comparisons, blue indicates ND → Q transitions, and light gray indicates other patterns. The dashed line indicates the 80% directional‐consistency threshold. Q, quantifiable; ND, non‐detectable or non‐quantifiable.

Detection‐state transitions supplemented several meta‐analytic findings. Terpinen‐4‐ol, (E)‐2‐octenal, and ethyl palmitate had three, two, and one ND‐to‐Q records, respectively, in addition to significant pooled increases. All three directional transitions for terpinen‐4‐ol were ND‐to‐Q, meeting the state‐consistency criterion and providing both quantitative and detection‐state support. The state directions for the other two compounds agreed with the meta‐analysis, but their classification as fermentation‐enriched was based primarily on the quantitative evidence because fewer than three transitions were available.

Ten additional compounds did not survive false‐discovery‐rate correction but showed agreement between their pooled direction and detection‐state records. Acetophenone, cedrol, methyl palmitate, (E)‐2‐nonenal, and 2‐methylnaphthalene tended to increase and had ND‐to‐Q records, whereas 1‐octen‐3‐ol, phenethyl alcohol, 2,2,6‐trimethylcyclohexanone, geraniol, and phytol tended to decrease and had Q‐to‐ND records. Because neither evidence stream met its prespecified decision criterion, these compounds were treated as trends and not assigned to the enriched or depleted categories.

Five methoxy‐substituted aromatic compounds, in addition to terpinen‐4‐ol, met the detection‐state consistency criterion. There were seven ND‐to‐Q records for 1,2‐dimethoxybenzene, five each for 1,2,3‐trimethoxybenzene and 1,2,4‐trimethoxybenzene, and three each for 1,4‐dimethoxybenzene and 4‐methoxystyrene, with no Q‐to‐ND transition for any of them. These five compounds were classified as fermentation‐enriched on the basis of detection‐state evidence. Their principal pattern was a transition from non‐detectable or non‐quantifiable before fermentation to quantifiable after fermentation, which cannot be represented adequately by Q‐to‐Q lnRRs.

No compound had at least three directional transitions with 80% or more in the Q‐to‐ND direction. Thus, detection‐state evidence alone did not identify a fermentation‐depleted compound; the only compound meeting the depletion criterion was linalool, on the basis of meta‐analysis.

Eight compounds had only sparse quantitative and detection‐state evidence: 1‐hexanol, camphor, (E,E)‐2,4‐decadienal, 2‐ethyl‐1‐hexanol, 2,2,6‐trimethyl‐6‐vinyltetrahydro‐2H‐pyran‐3‐ol, 3‐methyl‐1‐butanol, dihydro‐beta‐ionone, and isophytol. Each had only one or two Q‐to‐Q comparisons, all tending upward, and at least one concordant ND‐to‐Q record. These compounds were retained as exploratory candidates only.

Across both evidence streams, 15 compounds were classified as fermentation‐enriched. Ten were supported by significant quantitative increases after false‐discovery‐rate correction, with terpinen‐4‐ol also meeting the detection‐state criterion; five methoxy‐substituted aromatic compounds were supported primarily by consistent ND‐to‐Q transitions. Linalool was the only fermentation‐depleted compound. Pile fermentation therefore altered the volatile profile through both quantitative changes in pre‐existing compounds and transitions of selected compounds from non‐detectable to quantifiable.

### 3.4 Integrated Evidence and Change Types of Potentially Important Aroma Compounds

Among the 18 potentially important aroma compounds, alpha‐ionone, terpinen‐4‐ol, and (E)‐2‐octenal combined consistent post‐fermentation OAV evidence with significant pooled increases after false‐discovery‐rate correction (RRs of 2.79, 7.53, and 2.51, respectively). Terpinen‐4‐ol and (E)‐2‐octenal also had three and two ND‐to‐Q records. The methoxy‐substituted compounds 1,2‐dimethoxybenzene, 1,2,3‐trimethoxybenzene, and 1,2,4‐trimethoxybenzene had seven, five, and five ND‐to‐Q records, respectively, and OAV ≥ 1 in every evaluable study. These six compounds were therefore classified as fermentation‐enriched potentially important aroma compounds.

Linalool decreased significantly during pile fermentation (RR = 0.38, 95% CI 0.22–0.66) but retained OAV ≥ 1 in eight of nine evaluable studies, and was classified as a fermentation‐depleted potentially important aroma compound. The remaining 11 potentially important aroma compounds met the cross‐study OAV criterion but lacked either significant meta‐analytic evidence after correction or sufficient directional detection‐state evidence to assign a consistent change type. Integrating the three evidence streams thus distinguished post‐fermentation odor relevance from the direction of change during pile fermentation.

## 4 Discussion

From 1428 records, 11 studies were included in the synthesis. Eighteen compounds met the criterion of evaluation in at least five studies with OAV ≥ 1 in at least 80% of them. Meta‐analysis and detection‐state evidence identified 15 fermentation‐enriched compounds and one fermentation‐depleted compound. Integrating these changes with post‐fermentation OAV evidence prioritized alpha‐ionone, terpinen‐4‐ol, (E)‐2‐octenal, 1,2‐dimethoxybenzene, 1,2,3‐trimethoxybenzene, and 1,2,4‐trimethoxybenzene as fermentation‐enriched potentially important aroma compounds; linalool was a fermentation‐depleted potentially important aroma compound.

At the profile level, pile fermentation was characterized by an expansion in detectable volatiles. Among the 595 comparisons, 105 were ND‐to‐Q and only 37 Q‐to‐ND, consistent with reports that completed pile fermentation often yields a wider variety of detected volatiles than the starting material (Fang et al. 2024; P. Liu et al. 2022; Ma et al. 2021; Wen et al. 2023; W. Zhang, Cao, et al. 2021). An increase in the number of detectable compounds does not mean that all compounds rise in parallel; rather, it is compatible with the simultaneous release of bound precursors, production of microbial metabolites, and oxidation or degradation of pre‐existing volatiles.

The number of detected compounds and the total amount of volatiles are distinct attributes. Some industrial‐scale Qingzhuan and primary pile‐fermentation studies reported increases in total volatiles (Wu et al. 2026; H. Zhang, Wang, et al. 2021), yet changes differed by chemical class. Hu et al. (2021) observed decreases in alcohols and aldehydes but increases in ketones, olefins, and esters, whereas Q. Li et al. (2020) observed decreases in alcohols, aldehydes, and ketones but an increase in esters. Cultivar, maturity of the raw material, moisture, pile scale, fermentation endpoint, and analytical method may all contribute to these differences (Fang et al. 2024; Hu et al. 2021; Q. Li et al. 2020; Ma et al. 2021; W. Zhang, Cao, et al. 2021). We therefore did not pool total concentrations but evaluated within‐study compound‐level ratios and used detection‐state transitions to retain information from zeros and NDs.

The compound‐level synthesis exposed directional differences that are obscured by class totals. Significant increases occurred among terpene alcohols and their oxidation products, lipid‐derived aldehydes, esters, hydrocarbons, and carotenoid‐derived volatiles, whereas consistent ND‐to‐Q transitions were concentrated among methoxy‐substituted aromatic compounds. This pattern broadly agrees with reported increases in ketones, olefins, esters, and methoxy compounds (Hu et al. 2021; Q. Li et al. 2020; P. Liu et al. 2022), but compounds within the same class did not behave uniformly: terpinen‐4‐ol and alpha‐terpineol increased while linalool decreased, and aldehydes also followed compound‐specific trajectories. Class‐level summaries can therefore conflate distinct formation and depletion pathways.

Methoxy‐substituted aromatic compounds showed the clearest post‐fermentation appearance pattern. The three dimethoxy‐ or trimethoxybenzenes prioritized by integrated evidence repeatedly changed from ND to quantifiable and reached OAV ≥ 1 in every evaluable study; 1,4‐dimethoxybenzene and 4‐methoxystyrene also met the state‐based enrichment criterion. Molecular‐sensory studies have associated methoxybenzenes with the characteristic aged notes of dark tea (T. Li, Wei, et al. 2024; P. Liu et al. 2022; Pang et al. 2019). Comparisons among sterile moist‐heat treatment, natural microbial fermentation, and dominant‐fungus systems suggest that release, decarboxylation, and methylation of phenolic‐acid or phenolic precursors may jointly contribute to their formation, with fungal O‐methyltransferase‐related reactions representing a plausible step (T. Li, Wei, et al. 2024; X. Zhang et al. 2025). These mechanisms are consistent with the cross‐study pattern, but the specific substrates, methyl donors, and enzymes remain to be demonstrated directly in natural pile‐fermentation systems.

Lipid oxidation provides a coherent chemical basis for several aldehydes. (E)‐2‐Octenal, (Z)‐4‐heptenal, and safranal increased significantly, while (E,E)‐2,4‐heptadienal, heptanal, nonanal, octanal, benzaldehyde, phenylacetaldehyde, and hexanal showed consistent post‐fermentation OAV evidence. Linoleic and alpha‐linolenic acids can undergo lipoxygenase‐mediated peroxidation, followed by hydroperoxide‐lyase cleavage and secondary oxidation, to generate C6‐C9 aldehydes; warm, humid, and aerated pile conditions may also favor non‐enzymatic oxidation. A Qingzhuan‐tea study integrating lipidomics, volatilomics, model reactions, and stable‐isotope tracing further linked polyunsaturated‐fatty‐acid oxidation with the formation of (E,E)‐2,4‐heptadienal and (E)‐2‐octenal (P. Zheng, Feng, et al. 2025). The relative contributions of plant enzymes, microbial enzymes, and non‐enzymatic reactions at different fermentation stages nevertheless remain unresolved.

Terpene alcohols and their oxides showed related but asynchronous trajectories. Linalool decreased, whereas (Z)‐linalool oxide (furanoid), terpinen‐4‐ol, and alpha‐terpineol increased. Tea leaves contain glycosidically bound precursors of linalool and its oxides (D. Wang et al. 2000), and fungi can transform linalool into several oxygenated derivatives (Mirata et al. 2008). Linalool consumption and accumulation of selected oxidation products are therefore mechanistically compatible, but the present cross‐study evidence does not establish a direct precursor‐product relationship. Independent precursor pools, volatilization, thermal oxidation, and subsequent degradation may also contribute. Isotope‐labeled substrate tracing, quantification of glycosidic precursors, and enantiomer‐resolved analysis of oxidation products could discriminate among these possibilities.

Carotenoid cleavage may underlie the increases in alpha‐ionone, geranylacetone, and safranal. Enzymatic cleavage, autoxidation, and thermal degradation of carotenoids can generate ionones, safranal, and related volatiles (W. Liu et al. 2025; J. Wang et al. 2020). Alpha‐ionone increased significantly after false‐discovery‐rate correction and had OAV ≥ 1 in all nine evaluable studies. Beta‐ionone likewise had OAV ≥ 1 in all ten evaluable studies and ranked highly in relative concentration, yet its meta‐analysis did not remain significant after correction. These contrasting patterns may reflect differences in precursor composition, cleavage position, or product stability, and show that high post‐fermentation odor activity does not necessarily imply a consistent net increase during fermentation.

Ethyl palmitate and tridecane also increased significantly, but the limits of inference differ. Several studies reported higher total ester contents after pile fermentation (Hu et al. 2021; Q. Li et al. 2020; Wu et al. 2026), potentially arising from lipid hydrolysis, alcohol metabolism, and microbial esterase activity or esterification under moist‐heat conditions. In contrast, direct evidence for the formation pathway and sensory relevance of tridecane remains scarce. Statistical change or high abundance alone indicates association with pile fermentation; without consistent OAV evidence, it does not establish an important aroma role.

The OAV analysis separated changes during fermentation from potential odor relevance after fermentation. Some significantly increased compounds did not meet the cross‐study OAV criterion, whereas linalool decreased yet retained OAV ≥ 1 in eight of nine studies. Naphthalene ranked relatively low in concentration but had an apparent OAV ≥ 1 in all five evaluable studies. These observations illustrate the influence of odor threshold on screening, but thresholds measured in water cannot fully represent partitioning, adsorption, and compound interactions in dry tea, and OAV ≥ 1 cannot replace GC‐O, aroma‐recombination, or omission experiments (Han et al. 2026; Y. Li, Wang, et al. 2024; Pang et al. 2019; L. Wang et al. 2025; Zhao et al. 2024; X. Zheng, Xie, et al. 2025). Naphthalene and other compounds such as toluene, styrene, phthalates, and 2,4‐di‐tert‐butylphenol may also arise from environmental exposure, packaging, laboratory background, or analytical artifacts (Bai et al. 2025; Pan et al. 2025). Such compounds should therefore be treated as candidates requiring source verification, not accepted solely from threshold calculations as characteristic dark‐tea odorants.

The observed chemical changes are plausibly driven by both moist‐heat reactions and microbial metabolism. During natural pile fermentation, succession of Candida, Debaryomyces, Penicillium, Aspergillus, and Thermomyces has been associated with changes in alcohols, aldehydes, ketones, and esters (Guo et al. 2025; Hu et al. 2021). Inoculation studies further indicate that Bacillus species, Eurotium cristatum, Penicillium polonicum, and *Debaryomyces hansenii* differ in glycoside hydrolysis, lipid and amino‐acid metabolism, and oxidoreductive capacity (Gao et al. 2025; Huang et al. 2025; Xiao et al. 2022; C. Yang et al. 2025; X. Zhang et al. 2025). These studies provide biological hypotheses for methoxybenzene appearance, terpene‐alcohol transformation, and accumulation of lipid‐derived aldehydes. However, correlations do not establish causality, and single‐strain or co‐culture models do not reproduce the full ecological and physicochemical complexity of natural piles. Sterile moist‐heat controls, defined‐community add‐back experiments, enzyme‐activity measurements, and isotope tracing within a common processing system will be necessary to partition microbial and abiotic contributions.

The principal contribution of this review is to resolve conflicting reports at the level of individual compounds. To our knowledge, this is the first systematic review and meta‐analysis of volatile changes across primary pile fermentation of dark tea. Within‐study lnRR reduced the influence of measurement‐unit differences; Benjamini‐Hochberg correction addressed multiplicity, and zero or non‐detect records that could not yield lnRR were retained in a separate detection‐state analysis linked to post‐fermentation OAV consistency. This framework asks separately whether a compound changes in concentration, appears or disappears, and may reach odor‐relevant levels after fermentation, thereby avoiding inference from a single study, pooled heterogeneous absolute concentrations, or arbitrary imputation of NDs.

Several limitations remain. Individual compounds were represented by few independent comparisons, and 45 of 49 meta‐analyses had *I* ^2^ ≥ 75%; the data were therefore insufficient for stable meta‐regression by tea type, fermentation duration, endpoint, or analytical method, and pooled RRs are most informative for overall direction. Reporting of biological replicates, calibration, and non‐detect criteria was incomplete in some primary studies. Within‐study percentiles reduce differences in scale but cannot recover comparable absolute concentrations. OAV classification depends on threshold source and matrix and does not capture synergistic or masking interactions, while the criterion of at least three directional transitions with 80% agreement is a prespecified descriptive boundary rather than an inferential test. Finally, inoculation and model‐fermentation studies inform plausible mechanisms but cannot establish causation in natural piles. Future work should combine standardized, multi‐batch industrial sampling with targeted quantification of priority compounds and precursors, temperature and moisture monitoring, microbial succession, enzyme assays, GC‐O, recombination and omission experiments, and stable‐isotope tracing. Once sufficiently standardized datasets become available, machine‐learning models may help predict endpoint aroma from precursor, process, microbial, and volatile profiles; controlled fermentation and targeted enzyme studies could then translate validated pathways into quality‐control and product‐differentiation strategies.

Overall, pile fermentation does not increase all volatiles proportionally. It reshapes dark‐tea aroma through the appearance of new detectable compounds, depletion or accumulation of pre‐existing constituents, and multiple precursor‐conversion pathways. The post‐fermentation appearance of methoxy‐substituted aromatic compounds, accumulation of lipid‐derived aldehydes and carotenoid‐cleavage products, and opposing trajectories of linalool and selected oxidation products provide the clearest chemical patterns. Alpha‐ionone, terpinen‐4‐ol, (E)‐2‐octenal, and the three prioritized methoxy‐substituted aromatic compounds combine evidence of fermentation enrichment with consistent post‐fermentation odor relevance and warrant targeted quantification and sensory validation.

## 5 Conclusions

This systematic review and meta‐analysis integrated post‐fermentation odor activity, quantitative changes during primary pile fermentation, and detection‐state transitions to characterize aroma development in dark tea. Eighteen compounds met the predefined criterion for potentially important aroma compounds: β‐ionone, α‐ionone, benzaldehyde, (E,E)‐2,4‐heptadienal, heptanal, (E)‐2‐octenal, nonanal, octanal, phenylacetaldehyde, hexanal, terpinen‐4‐ol, cedrol, linalool, 1‐octen‐3‐ol, 1,2‐dimethoxybenzene, 1,2,3‐trimethoxybenzene, 1,2,4‐trimethoxybenzene, and naphthalene. These aroma compounds collectively contribute aged, woody, floral, sweet, herbal, and fatty notes to the characteristic aroma profile of dark tea.

Quantitative meta‐analysis and detection‐state analysis identified 15 fermentation‐enriched compounds: terpinen‐4‐ol, α‐terpineol, (Z)‐linalool oxide (furanoid), (Z)‐4‐heptenal, (E)‐2‐octenal, safranal, tridecane, ethyl palmitate, α‐ionone, geranylacetone, 1,2‐dimethoxybenzene, 1,2,3‐trimethoxybenzene, 1,2,4‐trimethoxybenzene, 1,4‐dimethoxybenzene, and 4‐methoxystyrene. Linalool was the only compound classified as fermentation‐depleted. By integrating fermentation‐related changes with post‐fermentation OAV consistency, α‐ionone, terpinen‐4‐ol, (E)‐2‐octenal, 1,2‐dimethoxybenzene, 1,2,3‐trimethoxybenzene, and 1,2,4‐trimethoxybenzene were identified as fermentation‐enriched potentially important aroma compounds. Linalool was classified as a fermentation‐depleted potentially important aroma compound because it decreased significantly during pile fermentation but retained OAV ≥ 1 in most evaluable studies. These findings indicate that the characteristic aroma of dark tea develops through compound‐specific formation, accumulation, transformation, and loss rather than a uniform increase in all volatile constituents.

The candidates identified here provide focused targets for mechanistic validation and fermentation control. Future studies should combine targeted quantification, GC–O, aroma recombination and omission tests, multi‐omics, enzyme assays, and stable‐isotope tracing to clarify the roles of O‐methyltransferases, glycosidases, lipid‐oxidation enzymes, carotenoid‐cleavage enzymes, and terpene‐converting enzymes. Once experimentally validated, selected enzymes or food‐grade microbial consortia could be optimized to promote desirable aged, woody, floral, and violet‐like notes while limiting undesirable odors. Standardized multi‐batch studies integrating processing parameters, microbial succession, aroma precursors, volatile markers, and sensory data could further support machine‐learning models for predicting aroma development and determining fermentation endpoints. A reduced marker panel comprising the prioritized fermentation‐enriched compounds and the fermentation‐depleted marker linalool may therefore facilitate rapid process monitoring, early identification of abnormal batches, and improved aroma consistency in industrial dark‐tea production.

## Author Contributions

**Shu Gong:** conceptualization, data curation, formal analysis, investigation, visualization, writing – original draft. **Jinwei Xu:** validation, writing – review and editing. **Guizhen Tang:** data curation, validation. **Binyu Mao:** investigation, visualization. **Qinmei Zhang:** validation, supervision, writing – review and editing. **Haimin Chen:** conceptualization, supervision, investigation, writing – review and editing.

## Funding

This work was supported by the Second Batch of Teaching Reform Projects for Higher Vocational Education in Zhejiang Province during the 14th Five‐Year Plan (jg20240178).

## Ethics Statement

The authors have nothing to report.

## Consent

The authors have nothing to report.

## Conflicts of Interest

The authors declare no conflicts of interest.

## Supporting information


**Data S1:** Supporting Information.

**Table S1:** fsn372499‐sup‐0002‐TableS1‐S9.xlsx. Database‐specific search strategies and study‐selection counts. Methods 2.1; Results 3.1; Figure 1.
**Table S2:** Study‐level methodological quality assessment. Methods 2.7; Results 3.1.
**Table S3:** Reports excluded after full‐text assessment. Results 3.1; Figure 1.
**Table S4:** Complete random‐effects meta‐analysis results. Methods 2.6; Results 3.3.1; Figure 4.
**Table S5:** Study‐level Q‐to‐Q effect sizes. Methods 2.6; Results 3.3.1.
**Table S6:** Compound‐level detection‐state summary. Methods 2.6.1; Results 3.3.2; Figure 5.
**Table S7:** Post‐fermentation within‐study concentration percentile summaries. Methods 2.4; Results 3.2; Figure 2.
**Table S8:** Post‐fermentation concentration, detection‐state, and OAV records. Methods 2.4–2.6.1; Results 3.2–3.3.
**Table S9:** Cross‐study OAV consistency. Methods 2.5; Results 3.2; Figure 3.

## Data Availability

The data that supports the findings of this study are available in the Supporting Information of this article.
