# Supplementary material for: Potentially Important Aroma Compounds in Dark Tea and Volatile Compound Changes During Primary Pile Fermentation: A Systematic Review and Meta‐Analysis
Source: Food Sci Nutr. 2026 Oct 28;14(11):e72499. doi: 10.1002/fsn3.72499 (PMC13717213; doi:10.1002/fsn3.72499)
Supplement: Supplementary file 1 [file FSN3-14-e72499-s001.docx]

**Manuscript: “Potentially important aroma compounds in dark tea and volatile compound changes during primary pile fermentation: A systematic review and meta-analysis”**

| **Section and Topic** | **Item #** | **Checklist item** | **Location where item is reported** |  |  |
| --- | --- | --- | --- | --- | --- |
| **TITLE** | | |  |  |  |
| Title | 1 | Identify the report as a systematic review. | Title page. |  |  |
| **ABSTRACT** | | |  |  | **ABSTRACT** |
| Abstract | 2 | See the PRISMA 2020 for Abstracts checklist. | Abstract. |  |  |
| **INTRODUCTION** | | |  |  |  |
| Rationale | 3 | Describe the rationale for the review in the context of existing knowledge. | Introduction, paragraphs 1–3. |  |  |
| Objectives | 4 | Provide an explicit statement of the objective(s) or question(s) the review addresses. | Introduction, final paragraph. |  |  |
| **METHODS** | | |  |  |  |
| Eligibility criteria | 5 | Specify the inclusion and exclusion criteria for the review and how studies were grouped for the syntheses. | Methods, Section 2.2; synthesis eligibility in Sections 2.4–2.6.1. |  |  |
| Information sources | 6 | Specify all databases, registers, websites, organisations, reference lists and other sources searched or consulted to identify studies. Specify the date when each source was last searched or consulted. | Methods, Section 2.1; Supplementary Data, Table S1. All sources were last searched on 18 August 2026. |  |  |
| Search strategy | 7 | Present the full search strategies for all databases, registers and websites, including any filters and limits used. | Methods, Section 2.1; complete database-specific strategies in Supplementary Data, Table S1. |  |  |
| Selection process | 8 | Specify the methods used to decide whether a study met the inclusion criteria of the review, including how many reviewers screened each record and each report retrieved, whether they worked independently, and if applicable, details of automation tools used in the process. | Methods, Section 2.1. |  |  |
| Data collection process | 9 | Specify the methods used to collect data from reports, including how many reviewers collected data from each report, whether they worked independently, any processes for obtaining or confirming data from study investigators, and if applicable, details of automation tools used in the process. | Methods, Section 2.3. |  |  |
| Data items | 10a | List and define all outcomes for which data were sought. Specify whether all results that were compatible with each outcome domain in each study were sought (e.g. for all measures, time points, analyses), and if not, the methods used to decide which results to collect. | Methods, Sections 2.3–2.6.2. |  |  |
|  | 10b | List and define all other variables for which data were sought (e.g. participant and intervention characteristics, funding sources). Describe any assumptions made about any missing or unclear information. | Methods, Sections 2.3–2.5; Table 1; Supplementary Data, Tables S2, S8, and S9. |  |  |
| Study risk of bias assessment | 11 | Specify the methods used to assess risk of bias in the included studies, including details of the tool(s) used, how many reviewers assessed each study and whether they worked independently, and if applicable, details of automation tools used in the process. | Methods, Section 2.7; Supplementary Data, Table S2. |  |  |
| Effect measures | 12 | Specify for each outcome the effect measure(s) (e.g. risk ratio, mean difference) used in the synthesis or presentation of results. | Methods, Section 2.6. |  |  |
| Synthesis methods | 13a | Describe the processes used to decide which studies were eligible for each synthesis (e.g. tabulating the study intervention characteristics and comparing against the planned groups for each synthesis (item #5)). | Methods, Sections 2.2 and 2.6. |  |  |
|  | 13b | Describe any methods required to prepare the data for presentation or synthesis, such as handling of missing summary statistics, or data conversions. | Methods, Sections 2.3–2.6.2; Supplementary Data, Tables S5, S8, and S9. |  |  |
|  | 13c | Describe any methods used to tabulate or visually display results of individual studies and syntheses. | Methods, Sections 2.4–2.6.2; Figures 2–5; Supplementary Data, Tables S4–S9. |  |  |
|  | 13d | Describe any methods used to synthesize results and provide a rationale for the choice(s). If meta-analysis was performed, describe the model(s), method(s) to identify the presence and extent of statistical heterogeneity, and software package(s) used. | Methods, Section 2.6 (DerSimonian–Laird random-effects model, inverse-variance weighting, Q, I², τ², and STATA 14). |  |  |
|  | 13e | Describe any methods used to explore possible causes of heterogeneity among study results (e.g. subgroup analysis, meta-regression). | No formal subgroup analysis or meta-regression was conducted because compound-specific study counts were small; heterogeneity is interpreted in Results Section 3.3.1 and the Discussion. |  |  |
|  | 13f | Describe any sensitivity analyses conducted to assess robustness of the synthesized results. | Not applicable. Zero/ND comparisons were not assigned positive values and were excluded from lnRR synthesis; they were summarized by detection state (Methods, Sections 2.6 and 2.6.2). |  |  |
| Reporting bias assessment | 14 | Describe any methods used to assess risk of bias due to missing results in a synthesis (arising from reporting biases). | No formal reporting-bias test was conducted because most compound-level syntheses contained few studies; this limitation is reported in the Discussion. |  |  |
| Certainty assessment | 15 | Describe any methods used to assess certainty (or confidence) in the body of evidence for an outcome. | No formal certainty-of-evidence framework was applied; study-level methodological quality is reported in Section 2.7 and Supplementary Data, Table S2, and limitations are discussed. |  |  |
| **RESULTS** | | |  |  |  |
| Study selection | 16a | Describe the results of the search and selection process, from the number of records identified in the search to the number of studies included in the review, ideally using a flow diagram. | Results, Section 3.1; Figure 1. |  |  |
|  | 16b | Cite studies that might appear to meet the inclusion criteria, but which were excluded, and explain why they were excluded. | Results, Section 3.1; all 39 reports excluded after full-text assessment and one primary reason per report are listed in Supplementary Data, Table S3. |  |  |
| Study characteristics | 17 | Cite each included study and present its characteristics. | Results, Section 3.1; Table 1. |  |  |
| Risk of bias in studies | 18 | Present assessments of risk of bias for each included study. | Results, Section 3.1; Supplementary Data, Table S2. |  |  |
| Results of individual studies | 19 | For all outcomes, present, for each study: (a) summary statistics for each group (where appropriate) and (b) an effect estimate and its precision (e.g. confidence/credible interval), ideally using structured tables or plots. | Supplementary Data, Tables S5 and S8; compound-level pooled results in Table S4 and Figure 4. |  |  |
| Results of syntheses | 20a | For each synthesis, briefly summarise the characteristics and risk of bias among contributing studies. | Results, Sections 3.1 and 3.3.1; Table 1; Supplementary Data, Table S2. |  |  |
|  | 20b | Present results of all statistical syntheses conducted. If meta-analysis was done, present for each the summary estimate and its precision (e.g. confidence/credible interval) and measures of statistical heterogeneity. If comparing groups, describe the direction of the effect. | Results, Sections 3.2–3.4; Figures 2–5; Supplementary Data, Tables S4–S9. |  |  |
|  | 20c | Present results of all investigations of possible causes of heterogeneity among study results. | No formal subgroup analysis or meta-regression was performed; observed heterogeneity is summarized in Results Section 3.3.1 and interpreted in the Discussion. |  |  |
|  | 20d | Present results of all sensitivity analyses conducted to assess the robustness of the synthesized results. | Not applicable; no positive-value imputation sensitivity analysis was required because zero/ND comparisons were excluded from lnRR synthesis and summarized descriptively. |  |  |
| Reporting biases | 21 | Present assessments of risk of bias due to missing results (arising from reporting biases) for each synthesis assessed. | Not formally assessed because compound-specific study counts were small; acknowledged in the Discussion. |  |  |
| Certainty of evidence | 22 | Present assessments of certainty (or confidence) in the body of evidence for each outcome assessed. | No formal certainty assessment was conducted; study-level quality and evidence limitations are reported in Section 3.1, Supplementary Data Table S2, and the Discussion. |  |  |
| **DISCUSSION** | | |  |  | **DISCUSSION** |
| Discussion | 23a | Provide a general interpretation of the results in the context of other evidence. | Discussion. |  |  |
|  | 23b | Discuss any limitations of the evidence included in the review. | Discussion, limitations paragraph. |  |  |
|  | 23c | Discuss any limitations of the review processes used. | Discussion, limitations paragraph; registration information in Methods, Section 2.1. |  |  |
|  | 23d | Discuss implications of the results for practice, policy, and future research. | Discussion and Conclusions. |  |  |
| **OTHER INFORMATION** | | |  |  | **OTHER INFORMATION** |
| Registration and protocol | 24a | Provide registration information for the review, including register name and registration number, or state that the review was not registered. | Methods, Section 2.1: the review was not prospectively registered. |  |  |
|  | 24b | Indicate where the review protocol can be accessed, or state that a protocol was not prepared. | Methods, Section 2.1: no prospectively registered protocol was available. |  |  |
|  | 24c | Describe and explain any amendments to information provided at registration or in the protocol. | Not applicable because the review was not registered and no prospective protocol was available. |  |  |
| Support | 25 | Describe sources of financial or non-financial support for the review, and the role of the funders or sponsors in the review. | Acknowledgments/Funding statement on the title page. |  |  |
| Competing interests | 26 | Declare any competing interests of review authors. | Conflicts of Interest statement. |  |  |
| Availability of data, code and other materials | 27 | Report which of the following are publicly available and where they can be found: template data collection forms; data extracted from included studies; data used for all analyses; analytic code; any other materials used in the review. | Data Availability Statement; Supplementary Data, Tables S1–S9. |  |  |

*From:*  Page MJ, McKenzie JE, Bossuyt PM, Boutron I, Hoffmann TC, Mulrow CD, et al. The PRISMA 2020 statement: an updated guideline for reporting systematic reviews. BMJ 2021;372:n71. doi: 10.1136/bmj.n71. This work is licensed under CC BY 4.0. To view a copy of this license, visit <https://creativecommons.org/licenses/by/4.0/>
